# N-6-Methyladenosine in Vasoactive microRNAs during Hypoxia; A Novel Role for METTL4

**DOI:** 10.3390/ijms23031057

**Published:** 2022-01-19

**Authors:** Daphne A. L. van den Homberg, Reginald V. C. T. van der Kwast, Paul H. A. Quax, A. Yaël Nossent

**Affiliations:** 1 Department of Surgery, Leiden University Medical Center, 2300 RC Leiden, The Netherlands; d.a.l.van_den_homberg@lumc.nl (D.A.L.v.d.H.); regi.vd.kwast@tum.de (R.V.C.T.v.d.K.); p.h.a.quax@lumc.nl (P.H.A.Q.); 2 Einthoven Laboratory for Experimental Vascular Medicine, Leiden University Medical Center, 2300 RC Leiden, The Netherlands; 3 Department for Laboratory Medicine, Medical University of Vienna, AT-1090 Vienna, Austria; 4 Department of Internal Medicine II, Medical University of Vienna, AT-1090 Vienna, Austria

**Keywords:** N-6-methyladenosine, m6A, microRNAs, vascular, fibroblasts, hypoxia, ischemia, METTL4, METTL3

## Abstract

N-6-methyladenosine (m6A) is the most prevalent post-transcriptional RNA modification in eukaryotic cells. The modification is reversible and can be dynamically regulated by writer and eraser enzymes. Alteration in the levels of these enzymes can lead to changes in mRNA stability, alternative splicing or microRNA processing, depending on the m6A-binding proteins. Dynamic regulation of mRNA m6A methylation after ischemia and hypoxia influences mRNA stability, alternative splicing and translation, contributing to heart failure. In this study, we studied vasoactive microRNA m6A methylation in fibroblasts and examined the effect of hypoxia on microRNAs methylation using m6A immunoprecipitation. Of the 19 microRNAs investigated, at least 16 contained m6A in both primary human fibroblasts and a human fibroblast cell line, suggesting vasoactive microRNAs are commonly m6A methylated in fibroblasts. More importantly, we found that mature microRNA m6A levels increased upon subjecting cells to hypoxia. By silencing different m6A writer and eraser enzymes followed by m6A immunoprecipitation, we identified METTL4, an snRNA m6A methyltransferase, to be predominantly responsible for the increase in m6A modification. Moreover, by using m6A-methylated microRNA mimics, we found that microRNA m6A directly affects downstream target mRNA repression efficacy. Our findings highlight the regulatory potential of the emerging field of microRNA modifications.

## 1. Introduction

MicroRNAs are a class of short (~22 nucleotides) non-coding RNA molecules, which regulate the translation of mRNAs into proteins. In cardiovascular diseases, the role of microRNAs as cardiovascular modulators has been well-established in multiple vascular remodelling processes [1]. It has been shown that microRNAs are involved in the different stages of cardiovascular disease, where they can play a role in the development of dyslipidaemia, vascular inflammation, hypertension and atherosclerotic lesion development and stability [2]. The progression of cardiovascular diseases can lead to insufficient blood flow through tissues, causing them to become ischemic and resulting in oxygen deprivation or hypoxia. MicroRNAs can also regulate the restoration of blood flow to these ischemic tissues by regulating the growth and maturation of blood vessels, a process called neovascularisation [3].

Like all RNAs, microRNAs are subject to post-transcriptional modifications [4]. One of the most abundant RNA modifications in eukaryotic cells is the conversion of adenosine into N6-methyladenosine (m6A). m6A is a reversible chemical modification that is regulated by methylating and demethylating enzymes [5]. The most well-known m6A ‘writers’, the m6A writer complex, composed of METTL3 and METTL14 (Methyltransferase Like 3 and 14), methylates nuclear RNAs in mammalian cells [6]. WTAP (Wilms’ tumour 1-associating protein) is one of several cofactors, which can interact with the writer complex to guide the complex to specific RNA targets [7]. However, other m6A writers exist too. METTL16, for example, can methylate coding and non-coding RNAs, including the snRNA U6 [8], while METTL4 has been identified as m6Am (2’O-ribose methylated m6A) methyltransferase of predominantly small nuclear RNAs [9]. m6A sites can also be demethylated by ‘erasers’ ALKBH5 (alkB homolog 5) and FTO (fat mass and obesity-associated protein), which allows for a more dynamic regulation of m6A, compared to most other RNA modifications [10,11].

Most of the m6A research has been focused on the methylation of mRNAs and other RNAs much longer than microRNAs. These studies have shown that m6A has important biological functions and can regulate numerous processes, including alternative splicing, mRNA decay and mRNA translation [12,13,14,15]. Most of these biological functions were found to be mediated through a group of m6A-‘reader’ proteins that specifically recognise the methylated adenosine [16,17]. The capacity of proteins to bind specifically to m6A marks has been exploited to map the location of m6A sites in mRNAs [12,13,14,15]. Interestingly, these studies demonstrated that adenosines within mRNAs that undergo m6A methylation were often found to be surrounded by a specific sequence motif, which can be described as [A/G/U][A/G]**A**C[A/C/U] or DRACH according to the IUPAC notation, in which the predicted methylated adenosine is displayed as **A **[16,17].

Recent studies have also demonstrated that m6A is dynamically regulated throughout life and is important for maintaining cardiovascular homeostasis [18,19]. Mathiyalagan et al. found that m6A is increased in failing mammalian hearts and in hypoxic cardiomyocytes [20]. Furthermore, Dorn et al. demonstrated that increased METTL3-dependant m6A causes spontaneous hypertrophy in mice, whereas METTL3 knockdown leads to maladaptive remodelling and signs of heart failure [21]. Additionally, increasing the expression of m6A eraser FTO in ischemic mouse hearts could attenuate the negative effects of m6A. Recently, it was discovered that the m6A landscape is differentially regulated in atherosclerotic lesions, showing that m6A plays a role in the development and progression of atherosclerosis [22]. Moreover, the m6A writer METTL3, as well as loss of the m6A eraser ALKBH5, were shown to have a positive effect on the promotion of angiogenesis [23,24]. These findings highlight a key role for m6A in ischemic cardiovascular disease and vascular remodelling.

Where these studies focused on longer RNAs, a very recent study by Chamorro-Jorganes et al. also shows the regulation of angiogenesis by METTL3 via m6A of two microRNAs, let-7e-5p and miR-18a-5p [25]. While m6A research has focused predominantly on mRNAs, several studies have demonstrated that m6A is important for microRNA biogenesis and functioning. Alarcon et al. found that primary microRNAs (pri-miRs) often contain METTL3-dependent m6A, which stimulates maturation of these pri-miR transcripts, mediated by the m6A reader HNRNPA2B1 [26,27]. Additionally, Berulava et al. demonstrated that many mature microRNAs also contain m6A in a human embryonic kidney cell line (HEK293) [28]. Interestingly, several microRNAs with a known vasoactive function were found to be methylated in HEK293 cells [28]. However, whether these mature vasoactive microRNAs are also subject to m6A methylation in vascular cells is unknown. 

As microRNA expression, and potentially modification patterns, are highly cell type-specific, we aimed to investigate whether vasoactive microRNAs are methylated in vascular cells and whether the m6A levels are actively regulated under pathological conditions such as hypoxia. Furthermore, we aimed to determine how m6A of vasoactive microRNAs may affect the regulation of the microRNAs’ mRNA targets involved in vascular remodelling and neovascularisation. To investigate the presence and regulation of m6A methylation in vasoactive microRNAs in vascular cells, we here selected a number of vasoactive microRNAs which were found to be m6A methylated in HEK293 cells and examined if they are also methylated in primary human arterial fibroblasts, which are known to have high microRNA expression levels [29], and we confirmed our finding in a human fibroblast cell line. Furthermore, we determined whether vasoactive microRNA m6A methylation is regulated in response to hypoxia and which m6A writer or eraser enzymes are responsible for m6A regulation in human fibroblasts, showing for the first time that METTL4 may be an important regulator of m6A in microRNAs. We then investigated whether m6A methylation affects the biogenesis of vasoactive microRNAs, using knockdown studies of m6A writers and erasers. Finally, we used synthetically m6A modified microRNA mimics to determine the effects of microRNA-m6A on target mRNA repression.

## 2. Results

### 2.1. The m6A Modification Is Present on Vasoactive microRNAs in Primary Arterial Fibroblasts

To investigate whether m6A is relevant to vasoactive microRNAs, we selected a subset of 19 vasoactive microRNAs to study in detail in fibroblasts (Table 1). This was done by cross-referencing the findings of Alarcon et al. [26] and Beruluva et al. [28] on microRNA m6A methylation in HEK293 cells, with several reviews on microRNAs in cardiovascular biology and disease [1,30,31,32,33]. The cross-referencing yielded a selection of 14 vasoactive microRNAs that appeared to be m6A methylated in one or both studies. We then added 5 additional vasoactive microRNAs (hsa-miR-329-3p [3,34,35], hsa-miR-494-3p [3,34,35], hsa-miR-487b-3p [3,34,35], hsa-miR-433-3p [36], and hsa-miR-155-5p [1]) that were not identified in either study using HEK293 to examine if this is consistent in fibroblasts. 

Then we performed an m6A pulldown to investigate whether these 19 vasoactive microRNAs were indeed methylated in primary human umbilical artery fibroblasts (HUAFs) by comparing their enrichment in the m6A immunoprecipitated fraction versus negative control IgG fraction, using rt/qPCR. We found that 16 of 19 mature microRNAs displayed a more than 25-fold enrichment in the m6A pulldown compared to the IgG pulldown (Table 1), indicating that m6A methylation of microRNAs is abundant in arterial fibroblasts. Strikingly, minimal or no enrichment of m6A was found for miR-223-3p and miR-136-5p, which were identified to be methylated in HEK293 by Beruluva et al. Furthermore, the additional five vasoactive microRNAs, which were not found in HEK293 cells, were highly enriched in the methylated fraction of HUAFs. These findings suggest that, like microRNA expression itself, microRNA m6A methylation may be cell-type specific.

We also measured the precursor microRNAs (pre-miRs) of the selected microRNAs. We could only detect 5 of the 19 pre-miRs in the m6A pulldown (Supplemental Table S2), which is consistent with previous findings that showed that pre-miRs are rapidly processed and are thus rather transient [37,38]. However, each of the five detected pre-miRs was enriched in the m6A fraction, indicating that m6A methylation is already present in the precursor stage. 

**Figure 1:**
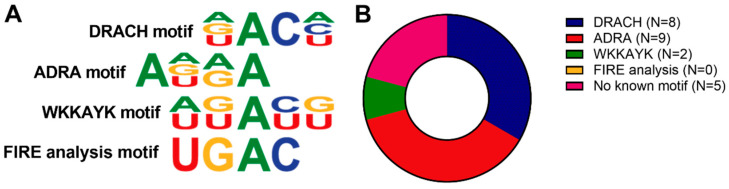
Predicted m6A motif in selected vasoactive microRNAs. (**A**) Visualisation of different m6A motifs described in the literature. (**B**) Schematic representation of m6A motifs found in the selection of vasoactive microRNAs.

As m6A is often found in or near specific sequence motifs, we analysed the sequences of the microRNAs for the DRACH-motif and found that it is present in only eight of the microRNAs (Figure 1, Supplemental Table S3). We also analysed three additional m6A motifs: A[A/G/U][A/G]**A**, or ADRA according to the IUPAC notation, the m6A motif specific for miRNAs; [A/U][G/U][G/U]**A**[C/U][G/U], or WKKAYK, the METTL3/WTAP motif, and UG**A**C, the m6A motif in pri-miRs discovered by FIRE analysis [7,27,28]. The METTL3/WTAP motif was found in two of the vasoactive microRNA sequences, while the FIRE analysis motif was not present at all. The ADRA motif was found in 9 out of the 19 vasoactive microRNAs. In five microRNAs, no m6A motif was found at all; however, DRACH and ADRA motifs were present when one mismatch was allowed. 

Taken together, each of the selected methylated microRNAs contains a complete or partial m6A methylation motifs DRACH or ADRA; however, there does not appear to be a strong link to any one motif in particular. 

### 2.2. Hypoxia Induces an Increase of m6A-Methylation in microRNAs

To ensure that changes in m6A methylation are not obscured by the biological variation inherent in primary human cell cultures, we conducted further experiments using a commonly used human fibroblast cell line (BJ cells). Like in HUAFs, the microRNAs with the lowest enrichment after m6A immunoprecipitation in BJ cells were also miR-136-5p and miR-223-3p (Supplemental Figure S1), indicating these cells provide a suitable model for primary fibroblasts. The 17 other selected vasoactive microRNAs were all enriched in the m6A immunoprecipitated fraction of these fibroblasts.

**Figure 2:**
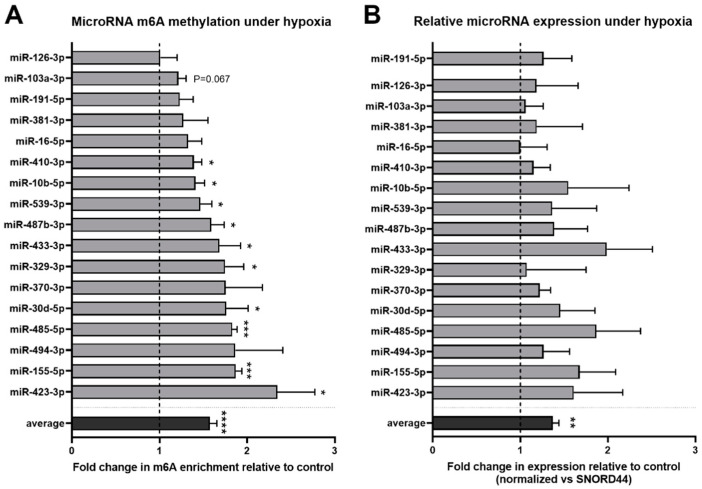
Effect of hypoxia on m6A methylation of selected vasoactive microRNAs. (**A**) Fold change in m6A enrichment after m6A immunoprecipitation on small RNAs under hypoxic conditions for 24 h relative to control conditions. Gray bars represent the average value measured per microRNA, whereas the black bar represents the average of all measured microRNAs. (**B**) Hypoxia-induced fold change in expression of mature vasoactive microRNAs relative to control conditions. MicroRNA expression was normalised to SNORD44. Statistically significant differences are indicated by * *p* ≤ 0.05, ** *p* ≤ 0.01, *** *p* ≤ 0.001, **** *p* ≤ 0.0001. Results represent three independent experiments.

Next, we investigated whether levels of vasoactive microRNA m6A methylation in fibroblasts are regulated in response to hypoxia. We found that mature microRNA m6A enrichment levels generally increased in vasoactive microRNAs under hypoxia as the overall average enrichment level was significantly increased by 1.6 fold (*p* < 0.0001) (Figure 2A). Individual microRNA m6A methylation levels of 10 microRNAs (miR-410-3p, miR-10b-5p, miR-539-3p, miR-487b-3p, miR-433-3p, miR-329-3p, miR-30d-5p, miR-485-5p, miR-155-5p, and miR-423-5p) were significantly increased in the m6A immunoprecipitation of hypoxic cells compared to that of control cells (Figure 2A). A similar trend (*p* < 0.075) was observed for one other microRNAs (miR-103a-3p). The overall average microRNA expression also increased slightly but significantly, by 1.3-fold, under hypoxia (*p* < 0.01), but no direct correlation was observed between the relative increase in microRNA methylation and the relative increase in microRNA expression (Figure 2B).

### 2.3. The Effect of Hypoxia on mRNA Levels of the m6A Machinery

**Figure 3:**
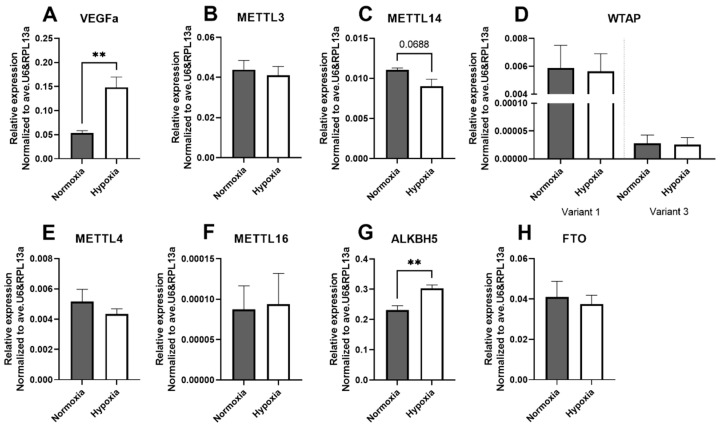
Expression of m6A machinery under hypoxic conditions. Relative mRNA expression levels of VEGFα (**A**), METTL3 (**B**), METTL14 (**C**), WTAP (**D**), METTL4 (**E**), METTL16 (**F**), ALKBH5 (**G**) and FTO (**H**) in BJ cells cultured under 24 h of hypoxic conditions. All mRNA levels were normalised to an average of the housekeeping genes U6 and RPL13a. Statistically significant differences are indicated by ** *p* ≤ 0.01. Results represent four independent experiments.

To investigate the potential relevance of microRNA m6A in ischemia, one of the hallmarks of most cardiovascular diseases, we measured m6A-writer and -eraser mRNA levels in fibroblasts that were cultured under hypoxic conditions for 24 h. We validated that hypoxia was successfully induced by demonstrating that hypoxic BJ cells had increased expression of vascular endothelial growth factor α (VEGFα), a gene well known to be induced by hypoxia signalling (Figure 3A) [39]. Hypoxia did not induce changes in mRNA levels of m6A writer complex proteins METTL3 and WTAP (Splice variants 1 and 3) or the m6A writers METTL4, METTL16 and m6A eraser FTO in BJ cells (Figure 3B,D–F,H). However, we did observe a significant upregulation in the mRNA levels of m6A-eraser ALKBH5 under hypoxia and a trend towards downregulation of mRNA levels of METTL14 (Figure 3C,G). Cells cultured under 48 h of hypoxia showed similar m6A writer and eraser levels as 24 h of hypoxia (Supplemental Figure S2); however, we did observe a significant upregulation of mRNA levels of the m6A writer METTL3 after 48 h of hypoxia, which was not observed after 24 h. These findings suggest the hypoxia-induced increase in mature microRNA m6A methylation, that was observed after 24 h of hypoxia, is not caused by changes in the expression of m6A-writer or -eraser proteins.

### 2.4. METTL4 as Novel m6A-Methyltransferase for Vasoactive microRNAs

**Figure 4:**
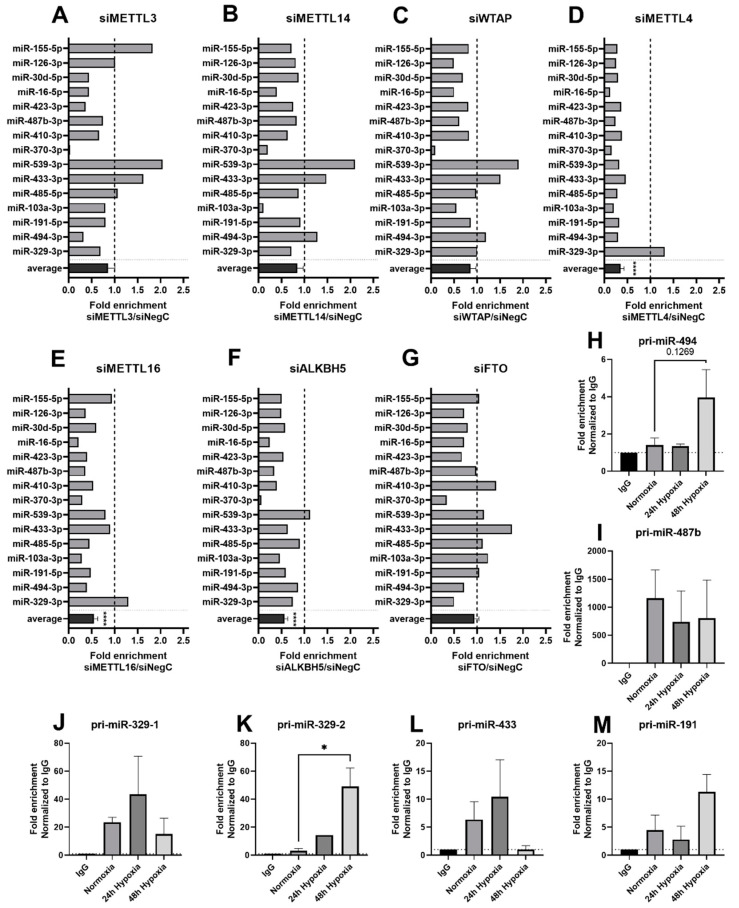
METTL4 as a novel m6A-methyltransferase for vasoactive microRNAs. m6A fold enrichment of mature microRNAs compared to negative control after knockdown of METTL3 (**A**), METTL14 (**B**), WTAP (**C**), METTL4 (**D**), METTL16 (**E**), ALKBH5 (**F**) and FTO (**G**) in BJ cells. Samples of multiple independent transfections were pooled to perform a single pulldown experiment. (**H**–**M**) Primary microRNAs bound to METTL4 in BJ cells cultured under normoxia or 24 or 48 h of hypoxia. Results represent three independent METTL4 RIP experiments. Statistically significant differences are indicated by * *p* ≤ 0.05.

To identify those m6A writers and readers responsible for m6A methylation of vasoactive microRNAs, we transfected fibroblasts with siRNAs against different writer and eraser enzymes (Supplemental Figure S2). We observed the most prominent change in overall average enrichment of m6A methylated microRNAs after knockdown METTL4 (Figure 4D). The m6A enrichment decreased for each individual microRNA (0.12–0.46-fold), except for miR-329-3p. The overall average enrichment level was significantly decreased by 0.3 fold (*p* < 0.0001). Knockdown of the m6A writer METTL16 also showed a significant decrease in overall average enrichment compared to the control (0.5 fold; *p* < 0.0001), however not all microRNAs showed a consistent decrease in m6A enrichment (Figure 4E). Knockdown of m6A writer complex proteins METTL3, METTL14 and WTAP or the eraser FTO did not result in significant changes in overall enrichment (Figure 4A–C). Unexpectedly, the knockdown of the eraser ALKBH5 decreased the overall average enrichment (0.5 fold; *p* < 0.0001). MicroRNA enrichment was not altered for siMETTL4, siMETTL16 or siALKBH5 in the total small RNA sample input used for the immunoprecipitation, indicating that the effects observed are specific and not merely caused by cellular stress induced by the transfection (Supplemental Figure S3D–F).

To investigate if METTL4 interacts with the vasoactive pri-miRs, we performed a RNA-binding protein immunoprecipitation (RIP) using an antibody against METTL4 on BJ cells cultured under normoxic or hypoxic conditions. The METTL4 pulldown samples were enriched for the selection of pri-miRs that were measured (Figure 4H–M). Moreover, cells cultured under either 24 or 48 h of hypoxia appeared to have a higher enrichment of pri-miRs in the METTL4 pulldown compared to cells cultured under normoxia. The fold enrichment of pri-miR-329-2 was significantly increased after 48 h of hypoxia (Figure 4K). pri-miR-494 and pri-miR-191 were also increased in the METTL4 pulldown under 48 h of hypoxia culture compared to normoxia, although not significantly (Figure 4H,M). pri-miR-329-1 and pri-miR-433 were increased after 24 h of hypoxia, not 48 h, however, these increases were not significant. With these results, we confirmed METTL4 as a novel m6A-methyltransferase for these vasoactive microRNAs.

### 2.5. Pri-, Pre-miR and Mature microRNA Expression after Knockdown of METTL4, METTL3 ALKBH5, or FTO

**Figure 5:**
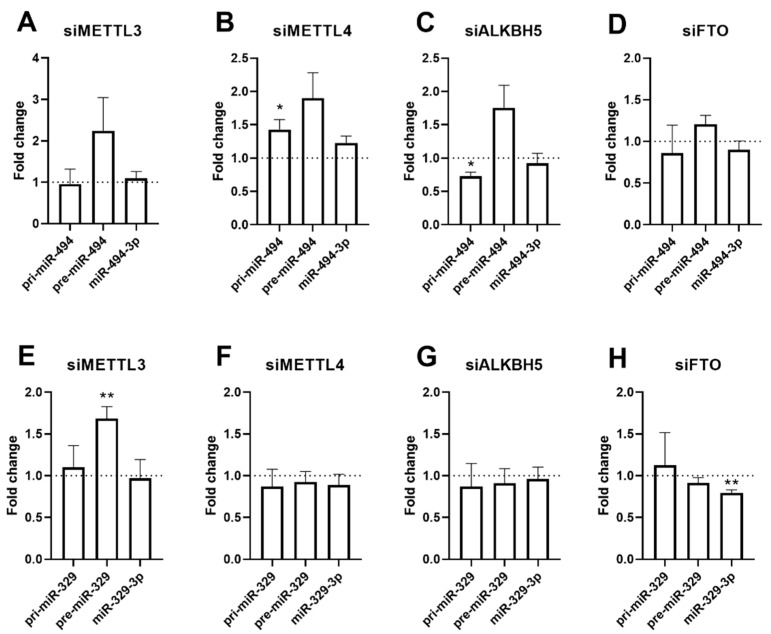
microRNA processing. The levels of primary (pri-), precursor (pre-), and mature microRNA-329-3p and -494-3p after siRNA-knockdown of METTL3, METTL4, ALKBH5 and FTO in BJ cells (**A**–**H**). Expression levels were normalised to the housekeeping gene U6. Statistically significant differences are indicated by * *p* ≤ 0.05, ** *p* ≤ 0.01. Data represent three independent experiments.

Several studies have indicated that m6A methylation of microRNA precursor transcripts, especially the pri-miRs, facilitates the biogenesis of microRNAs [25,26,27]. Therefore, we measured precursor microRNA expression of two model microRNAs, miR-494-3p and miR-329-3p, after siRNA mediated knockdown of METTL3, METTL4, ALKBH5 or FTO. We did observe minor changes in several microRNA processing intermediate products (Figure 5). We observed a significant upregulation of pri-miR-494 and pre-miR-329 after knockdown of m6A writers METTL4 and METTL3, respectively (Figure 5B,E). Knockdown of m6A erasers ALKBH5 and FTO yielded a significant decrease in pri-miR-494 or miR-329-3p (Figure 5C,H). However, as we anticipated, to find an accumulation of the prim-miR and depletion of the pre- and mature microRNA upon knockdown of METTL3 and/or METTL4, and the opposite upon knockdown of ALKBH5 and/or FTO, these findings suggest that effects on processing are not the determining mechanism-of-action in m6A of vasoactive microRNAs. 

### 2.6. Altered Target Repression of [m6A]Methylated Mature microRNA-494-3p

**Figure 6:**
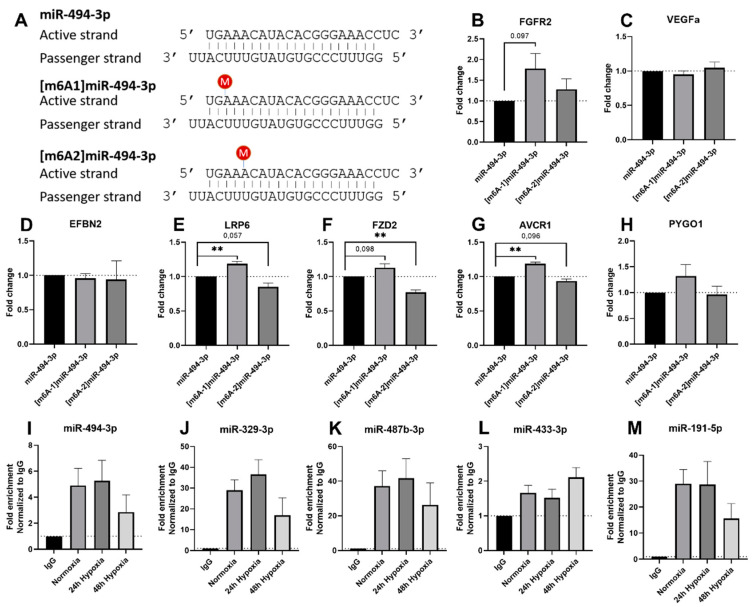
Functional effects of m6A-methylation of vasoactive microRNAs. (**A**) Sequences of microRNA mimics of methylated and unmethylated miR-494-3p. (**B**–**H**) mRNA expression of previously confirmed targets of miR-494-3p was measured after microRNA mimic transfections in BJ cells. mRNA targets were normalised to the housekeeping gene RPL13a. Data represent three independent experiments. (**I**–**M**) MicroRNAs bound to AGO2 in BJ cells cultured under normoxia or 24 or 48 h of hypoxia. Results represent three independent AGO2 RIPs. Statistically significant differences are indicated by ** *p* ≤ 0.01.

As it was previously reported that m6A methylation in mature microRNAs can influence target repression [40,41], we ordered synthetically methylated and unmethylated microRNA mimics of microRNA miR-494-3p to study the effects on its downstream targets. We chose the location of the m6A methylation based on predictions of the m6A motifs (Figure 1) and the location of the adenosine residues within the seed sequence, i.e., nucleotides 2–7 from the 5′-end. The sequences of the microRNA mimics are visualised in Figure 6A, the first and third adenosine are synthesised as m6A in the methylated mimics, respectively. After mimic transfection, we measured the mRNA expression of confirmed downstream targets of miR-494-3p. FGFR2, VEGFa and EFBN2 have previously been validated as direct targets of miR-494-3p using luciferase assays, while LRP6, FZD2, AVCR1 and PYGO1 have been identified as targets of miR-494-3p as predicted by Targetscan.org (v7.2) and rt/qPCR [3,42]. Transfection of [m6A1]miR-494-3p resulted in a significant increase in mRNA expression of LRP6 and AVCR1 and a trend towards significance (*p* < 0.1) of FGFR2 and FZD2 compared to its unmethylated equivalent, indicating a decrease in target repression after [m6A1]miR-494-3p transfection (Figure 6B,E–G). Interestingly, we observed an opposite effect when cells were transfected with [m6A2]miR-494-3p. mRNA expression of FZD2 was significantly decreased compared to the unmethylated miR-494-3p mimic, and a similar trend was observed for LRP6 and AVCR1 (Figure 6B–H).

As Briand et al. showed that m6A-methylated miR-200b-3p was recruited less effectively into the RISC complex [41], we were interested in the decreased target repression that we observed following mimic transfection could be explained, at least in part, by decreased RISC loading. Therefore, we performed an Argonaute RISC Catalytic Component 2 (AGO2) RIP on BJ cells cultured under normoxic or hypoxic conditions, as we observed an increase of mature microRNA m6A levels under hypoxia. We did not observe significant changes in fold enrichment of miR-494-3p, miR-329-3p, miR-487b, miR-433-3p and miR-191-5p in the AGO2 pulldown between the normoxic and hypoxic conditions. However, there appeared to be a trend in decreased fold enrichment in the AGO2 pulldown of miR-494-3p, miR-329-3p, miR-487b and miR-191-5p after 48 h of hypoxia (Figure 6I–M), which would correspond to a decrease in RISC loading of these microRNAs under hypoxia. 

## 3. Discussion

In this study, we show that of the 19 microRNAs investigated, at least 16 contained m6A in both primary human arterial fibroblasts and in a human fibroblast cell line, demonstrating that vasoactive microRNAs are commonly m6A methylated. We observed a different microRNA methylation pattern in our fibroblasts than previously found in HEK293, indicating that, like microRNA expression itself, microRNA m6A methylation may be cell-type specific. We found that mature microRNA m6A levels increased under hypoxia and that methylation appeared to depend mostly on the m6A writer METTL4. Finally, we demonstrate that m6A of vasoactive microRNAs does not affect microRNA processing but rather appears to reduce RISC-loading and alter target mRNA repression.

Although previous studies had already demonstrated that mRNA m6A methylation is dynamically regulated in response to ischemia and hypoxia [18,20,21], our data demonstrate that microRNA m6A levels are also dynamically regulated in direct response to hypoxia. Most of these studies suggest that the degree of mRNA m6A methylation depends directly on levels of m6A writers or erasers. Therefore, we were surprised to find that, except for a slight decrease in METTL14 mRNA expression, no changes in m6A writer mRNA expression was observed after 24 h of hypoxia, suggesting the hypoxia-induced microRNA methylation is not facilitated by changes in writer complex abundance. 

Besides a downregulation of the writer METTL14, we also observed an upregulation of the eraser ALKBH5 under hypoxia. ALKBH5 was shown to be a target of HIF1α (Hypoxia Inducible Factor 1 Subunit Alpha), which provides a potential mechanism for its hypoxia-inducibility [43]. In a recent study in a breast cancer cell model, Zhang et al. showed that the upregulation of ALKBH5 under hypoxia was critical for the demethylation and stabilisation of NANOG mRNA [44]. Another study corroborated this finding by showing a decreased total m6A level in mRNA mediated by the upregulation of ALKBH5 under hypoxia [45]. However, in our study, we observed an increase in m6A methylation of microRNAs rather than a decrease, suggesting that it is unlikely that ALKBH5 targets any of the examined microRNAs. This is supported by previous findings that murine ALKBH5 generally targets a very different type of RNA than microRNAs, namely the 3’UTRs of longer mRNAs [10,44]. 

When aiming to determine which writer or eraser is responsible for m6A methylation of vasoactive microRNAs in fibroblasts, we showed that specifically, knockdown of METTL4 resulted in a general decrease in m6A. In contrast, knockdown of the individual m6A writer complex proteins (METTL3, METTL14, WTAP) showed no change in overall average m6A enrichment. This suggests that m6A methylation of mature vasoactive microRNAs is not predominantly regulated by METTL3 but instead by METTL4. Even though we did not observe changes in mRNA expression of METTL4 induced by hypoxia, we did measure a higher enrichment of vasoactive primary microRNAs bound to METTL4 under hypoxia compared to normoxia. It has been shown previously that the rate of m6A methylation can be regulated without changes in writer expression, as SUMOylation of the writer METTL3 was demonstrated to activity modulate its activity [46]. It is likely that the m6A methylation by the different m6A writers follows a microRNA specific pattern as Briand et al. showed that miR-200b-3p was m6A methylated by METTL3 [41] and vascular microRNAs let-7e and miR-18a-5p were as well [25]. The notion that different microRNAs are modified by different RNA binding proteins is not new. For example, we demonstrated previously that A-to-I editing of vasoactive microRNAs by either ADAR1 or ADAR2 is also microRNA specific [47].

METTL4 was recently discovered to catalyse the modification m6Am in U2 snRNA by N-6-methylation of 2’-O-methyladenosine (Am) [9]. It has been discovered that both m6A and m6Am are present in microRNAs [48]. Moreover, in a previous study, we showed that one of the vasoactive microRNAs measured here, miR-487b-3p, also carries an Am modification [49]. As the anti-m6A antibody cannot distinguish between m6A and m6Am, the precise combination of modifications and their location on each vasoactive microRNA should be identified with mass spectrometry in follow-up studies [48]. This same microRNA, miR-487b-3p, also carries another modification, namely A-to-I editing [49]. Like m6A and Am, A-to-I editing increased under ischemia and contributed to a pro-angiogenic phenotype of the microRNA. Interestingly, in vitro studies have shown that m6A of adenosine can almost completely prevent A-to-I editing of that adenosine, suggesting RNA m6A methylation could, in fact, help regulate A-to-I editing [50]. Xiang et al. demonstrated that m6A is indeed negatively correlated with A-to-I editing in mRNAs, although m6A and editing locations often did not overlap [51]. As we observe increases in all three modifications, Am, A-to-I and m6A in a single microRNA under hypoxia, this microRNA would be a highly interesting target for future investigations into the interplay between microRNA modifications.

It is generally assumed that m6A methylation occurs on a microRNA’s primary transcript due to the nuclear localisation of the m6A methylation complex [18]. Therefore the observed differences in mature microRNA m6A could be caused by changes in m6A-mediated microRNA processing rather than by changes in m6A methylation rates. Recently, it was discovered that the m6A-reader protein HNRNPA2B1 facilitates the processing of primary into mature microRNAs in an m6A-dependant manner. Another study identified an m6A reader protein, NF-κB associated protein (NKAP), that has a similar function to HNRNPA2B1 [52]. However, in human fibroblasts, we found no evidence of changes in microRNA processing rates. All previous studies have shown that m6A methylated mature microRNAs can have decreased mRNA silencing activity [40,41], we used m6A methylated microRNA mimics to study the effect on target repression of the vasoactive microRNA miR-494-3p. Interestingly, we observed a decreased target repression when the m6A mark was located on the 3rd nucleotide of the mature microRNA, but an increased target repression with the m6A mark on the 5th nucleotide, both located within the seed sequence of the microRNA. Konno et al. demonstrated a structural change in the RISC complex because of m6A-methylated miR-17-5p or miR-let-7a-5p and a smaller space around the mRNA recognition site based on 3D modelling [40]. It would be interesting to investigate whether the m6A-methylated mimics that we used in this study lead to a different structural change of the RISC complex that could explain the observed difference in effect on target repression. Moreover, m6A was found to block noncanonical A:G base pairing, which can decrease mRNA silencing activity [40,53,54]. 

Besides decreased target recognition, Briand et al. showed decreased RISC loading of m6A-methylated miR-200b-3p [41]. In this study, we did not observe significantly decreased interaction of our vasoactive microRNAs with RISC-associated AGO2 after hypoxia. However, there did appear to be a trend towards a decrease in microRNAs bound to AGO2 after 48 h of hypoxia., which would concur with a decrease in RISC-loading of m6A-methylated microRNAs. However, before drawing any conclusions on the function of m6A-methylated miR-494-3p, or other vasoactive microRNAs, it would be crucial to elucidate the exact location of the methylated adenosine residues. 

In conclusion, we demonstrate here that m6A methylation of vasoactive microRNAs is highly abundant in both primary human arterial fibroblasts and in a human fibroblast cell line. Furthermore, we show that the methyltransferase METTL4 is likely the main regulator of m6A of vasoactive microRNAs in human fibroblasts. Importantly, we demonstrate that vasoactive microRNA m6A methylation increases under hypoxic conditions, highlighting that, similar to mRNA m6A, microRNA m6A methylation is dynamically regulated. We further show that m6A methylation of the vasoactive microRNA miR-494-3p influences downstream target mRNA repression. As the m6A methylation of mature microRNAs appears to directly affect a microRNA’s gene silencing efficacy, future studies into the functional consequences of microRNA m6A in the vasculature are imperative.

## 4. Materials and Methods

### 4.1. Cell Culture

Human Umbilical Arterial Fibroblasts (HUAFs) were isolated and cultured in DMEM GlutaMAX (Thermo Fisher Scientific, Breda, The Netherlands) at 37 °C under 5% CO_2_, as described before [3]. Human skin fibroblasts, BJ (ATCC, CRL-2522), were obtained from American Type Culture Collection (ATCC) and cultured at 37 °C under 5% CO_2_ using Minimum Essential Medium Eagle (MEM; Sigma-Aldrich, Amsterdam, The Netherlands), supplemented with stable GlutaMAX™ (Thermo Fisher Scientific), 10% foetal bovine serum and Penicillin/Streptomycin. The medium was refreshed every 2–3 days. Cells were passed at 80–90% confluency using trypsin-EDTA. For hypoxic conditions, HUAFs and BJ cells were first seeded and cultured at 37 °C with 20% O_2_ and 5% CO_2_ for 24 h, and subsequently cultured at 37 °C with 1% O_2_ and 5% CO_2_ for the remainder of the experiment. 

### 4.2. rt/qPCR

Total RNA was isolated using Trizol reagent (Thermo Fisher Scientific) following the manufacturer’s protocol. RNA concentration and purity were measured using Nanodrop (Nanodrop Technologies, Wilmington, DE, USA). A High Capacity cDNA kit (Thermo Fisher Scientific) was used for the reverse transcription reaction to synthesise cDNA of the extracted RNA. rt/qPCR of mRNAs, pri- and pre-microRNAs was performed using Quantitect SYBR green (Qiagen), and the reactions were run on a Viia7 system (Thermo Fisher Scientific, for primer sequences, see Supplemental Table S1). TaqMan MicroRNA assays (Thermo Fisher Scientific) were used for rt/qPCR of microRNAs, consisting of a reverse transcription reaction using a specific microRNA primer and a qPCR reaction with TaqMan probes. For normalisation, we used the housekeeping genes RPL13a, U6 and SNORD44. To obtain the fold change of RNA expression, 2ΔΔCt values were calculated using the Ct values obtained with rt/qPCR. Results are displayed as a relative expression in experimental conditions relative to expression in control conditions. 

### 4.3. m6A Immunoprecipitation

The High Pure miRNA Isolation Kit (Sigma-Aldrich) was used to isolate separate fractions of small (<100 nucleotides) and large RNAs (>100 nucleotides) according to the manufacturer’s instructions. Immunoprecipitation (IP) of the small RNA fraction was performed using the EZMagna RIP kit (Millipore, Sigma Aldrich) as described previously [55]. To immunoprecipitate RNA-containing m6A, 5 µg of affinity-purified anti-m6A polyclonal antibody (Synaptic Systems, 202 003) or negative control anti-rabbit IgG (Millipore, Sigma Aldrich, PP64B) were coupled to magnetic beads, and 5 µg small RNA were incubated with these antibodies in immunoprecipitation (IP) buffer overnight at 4 °C. Before IP, 10% of RNA was taken out to serve as input reference. After overnight incubation, RNA bound to the magnetic beads was immobilised using magnets, and unbound RNA was washed away. Then, bound RNA was eluted with proteinase K buffer, consisting of RIP wash buffer, 10% SDS and Proteinase K as supplied by the manufacturer. Immunoprecipitated RNA was isolated according to standard protocol using Trizol LS reagent (Thermo Fisher Scientific). 

cDNA synthesis for pre-microRNA quantification was performed using SuperScript™ IV VILO™ Master Mix (Invitrogen) and subjected to rt/qPCR using primers against a selection of pre-microRNAs (for primer sequences, see Supplemental Table S1). MicroRNA quantification was performed using TaqMan microRNA assays, as described above.

### 4.4. m6A Motif Analysis

Transcriptome-wide studies, which used m6A immunoprecipitation on fragments of total RNA, showed that m6A was most prevalent in a certain sequence motif, which can be described as [AGU][AG]AC[ACU] or with IUPAC code DRACH [16,17], A[A/G/U][A/G]**A**, or ADRA [28], [A/U][G/U][G/U]**A**[C/U][G/U] or WKKAYK [7], and UG**A**C [27]. To analyse our precursor and microRNA sequences for this motif, we used the sequences of our vasoactive microRNAs from miRBase (http://www.miRbase.com, accessed on 1 June 2020) and analysed these sequences for the different motifs in R Studio (RStudio Desktop 1.2.5001). 

### 4.5. siRNA-Mediated Knockdown of m6A Machinery

BJ cells were seeded in 12 wells flat bottomed plates at a concentration of 70.000 cells/mL and cultured overnight at 37 °C. KN-93 (Sigma Aldrich) was added to the culture media (MEM, 10% FBS and 1% P/S) at a concentration of 10 µM. After 48 h, cells were washed with PBS, and a culture medium without serum or PenStrep was added. Then, the cells were transfected with a siRNA (small interfering RNA) against METTL3, METTL14, WTAP, METTL4, METTL16, ALKBH5 or FTO by using Lipofectamine RNAiMax (Thermo Fisher Scientific) according to the manufacturer’s protocol. Predesigned siRNAs (Thermo Fisher Scientific; WTAP (SASI_Hs01_00039282), ALKBH5 (SASI_Hs02_00350577), METTL3 (SASI_Hs01_00044317)) and MISSION esiRNAs (Sigma Aldrich; METTL14 (EHU038041), METTL4 (EHU083031), METTL16 (EHU002191), FTO (EHU092071)) were used with a final concentration of 75 nM siRNA or 30 nM esiRNA, respectively. A previously validated scrambled sequence siRNA was used as a negative control (5′-UCUCUCACAACGGGCAU(dT)(dT)-3′) [56]. Cells were transfected for 24 h, after which they were washed with PBS and lysed with subsequent m6A immunoprecipitation or RNA isolation, as described above. 

### 4.6. MicroRNA Mimic Transfection

m6A-methylated and unmethylated microRNA mimics were synthesised by Microsynth (Microsynth AG, Balgach, Switzerland) as separate oligonucleotides for the active and passenger strand. microRNA mimics for miR-494-3p were designed using the mature sequence of the microRNA and an AU-overhang in the passenger strand; the sequences are given in Supplementary Table S4. The active and passenger stand oligonucleotides were annealed at 95 °C for 5 min and allowed to cool down to room temperature slowly. BJ cells were seeded at 70,000 cells/mL in a 12 wells flat bottomed plate. The next day, the cells were transfected with the microRNA mimics at a final concentration of 0.5 nM for 24 h using Lipofectamine RNAiMax (Invitrogen). Then, cells were washed with PBS and lysed using Trizol for further RNA isolation, as described above.

### 4.7. RNA-Binding Protein Immunoprecipitation 

RNA binding protein immunoprecipitation (RIP) was performed using the EZMagna RIP kit (Millipore, Sigma Aldrich) according to the manufacturer’s protocol. In brief, BJ cells, one T175 flask was used per RIP reaction, cultured under normoxic or hypoxic conditions were washed with cold PBS twice, trypsinised and centrifuged for 5 min at 800 rpm to form a pellet. Cell pellets were resuspended in 0.1% formaldehyde to crosslink the RNA-protein interactions and incubated on ice for 30 min. After crosslinking, cell pellets were resuspended in complete RIP lysis buffer and incubated for 60 min on ice. To immunoprecipitate RNA bound to either AGO2 or METTL4, 5 µg of antibodies against AGO2 (Thermo Fisher Scientific, MA5-23515) or METTL4 (Abbexa LTD, abx235140) or control IgG antibodies of the respective species were conjugated to magnetic beads. Cell lysates were incubated together with antibody bound magnetic beads overnight at 4 °C rotating. Prior to incubation with magnetic beads, 10% of the cell lysate was kept separate as an input reference for the immunoprecipitation. The next day, samples were washed 6 times with RIP washing buffer and treated with proteinase K for 30 min at 55 °C while shaking to digest the protein. After incubation, samples were placed in a magnetic separator, and the supernatant was aspirated and transferred to a new tube. Samples were further diluted to a final volume of 250 µL in RNAse free water. RNA was isolated using TRIzol LS Reagent (Thermo Fisher Scientific) for liquid solutions. RNA concentration and purity were measured on the Nanodrop (Nanodrop Technologies).

cDNA synthesis for mRNA and pri-microRNA quantification was performed using SuperScript™ IV VILO™ Master Mix (Invitrogen). MicroRNA quantification was performed using TaqMan microRNA assays, as described above.

## 5. Statistical Analysis

Statistical analysis was performed using Student’s *t*-tests, and graphs were built using GraphPad Prism software (v9; GraphPad). Data are represented as mean ± standard error of mean (SEM). For the m6A immunoprecipitations, the total average of fold enrichment of the different microRNAs was calculated, and one-sample *t*-tests were performed to investigate the difference compared to control. *p*-values below 0.05 were considered statistically significant, and *p* < 0.1 was considered a trend.

## Figures and Tables

**Table 1:** Enrichment of selected vasoactive microRNAs in the m6A immunoprecipitation fraction of primary human umbilical arterial fibroblasts.

#	Selected microRNAs	Previous Findings Using HEK293 Cells		m6A Immunoprecipitation Using HUAFs (m6A IP/IgG IP)
		m6A Affects microRNA Biogenesis [26]	Mature microRNA m6A Methylated [28]	Mature microRNA Fold Enrichment
1	hsa-miR-10b-5p	√	√	67,077.50
2	hsa-miR-103a-3p	√	√	46,305.86
3	hsa-miR-485-5p	√	√	30,523.08
4	hsa-miR-423-5p	√	√	18,363.39
5	hsa-miR-30d-5p	√	√	12,245.17
6	hsa-miR-329-3p			3627.12
7	hsa-miR-126-3p	√		3159.09
8	hsa-miR-155-5p			3116.59
9	hsa-miR-16-5p	√	√	2782.99
10	hsa-miR-494-3p			1806.96
11	hsa-miR-487b-3p			1456.64
12	hsa-miR-381-3p	√		486.71
13	hsa-miR-191-5p	√	√	294.84
14	hsa-miR-370-3p		√	271.33
15	hsa-miR-410-3p		√	137.55
16	hsa-miR-433-3p			45.64
17	hsa-miR-539-3p		√	5.93
18	hsa-miR-223-3p		√	3.34
19	hsa-miR-136-5p		√	0.018

MicroRNA enrichment in the m6A immunoprecipitated fraction (m6A IP) relative to the negative control fraction (IgG IP). The fold enrichment was calculated using the averages of each fraction after measuring them in triplicate. HUAF, human umbilical arterial fibroblasts.

## Data Availability

All data are reported in the manuscript.

## Supplementary Materials

The following supporting information can be downloaded at: https://www.mdpi.com/article/10.3390/ijms23031057/s1.
