# Supplementary material for: N-6-Methyladenosine in Vasoactive microRNAs during Hypoxia; A Novel Role for METTL4
Source: Int J Mol Sci. 2022 Jan 19;23(3):1057. doi: 10.3390/ijms23031057 (PMC8835077; doi:10.3390/ijms23031057)
Supplement: Supplementary file 1 [file ijms-23-01057-s001.zip › ijms-1453119-supplementary.pdf]

International Journal of *Molecular Sciences*

Supplementary Materials

N-6-Methyladenosine in Vasoactive microRNAs during Hypoxia; A Novel Role for METTL4

Daphne A.L. van den Homberg, Reginald V.C.T. van der Kwast, Paul H.A. Quax and A. Yaël Nossent

Supplementary Table S1. Primer sequences.

Name	Primer type	Sequence
mRNA primers		
hsa-METTL3 F	Forward	ACACGTGGAGCTCTATCCAG
hsa-METTL3 R	Reverse	CGGAAGGTTGGAGACAATGC
hsa-METTL14 F	Forward	AGAGTGTGTTTACGAAAATGGGG
hsa-METTL14 R	Reverse	GATCCCCATGAGGCAGTGTT
hsa-METTL4 F	Forward	TGCCAGCAAAGTAATGAAAAGGA
hsa-METTL4 R	Reverse	CAGCTCCCTGATCTTTGTATGGT
hsa-METTL16 F	Forward	CCTCGCAACAGAAGTGGATG
hsa-METTL16 R	Reverse	GAGCATCCATCAGGAGTGTCT
hsa-WTAP var1 F	Forward	ACACGTGGAGCTCTATCCAG
hsa-WTAP var1 R	Reverse	CGGAAGGTTGGAGACAATGC
hsa-WTAP var3 F	Forward	CTCCCTTCACCTTTCCTCTCCTG
hsa-WTAP var3 R	Reverse	TGAAGTCTGTTTCACTCAATCGAAC
hsa-ALKBH5 F	Forward	ACCACCCAGCTATGCTTCAG
hsa-ALKBH5 R	Reverse	GCCGGTTCTCTTCCTTGTCC
hsa-FTO F	Forward	TCTGACCCCCAAAGATGATGAA
hsa-FTO R	Reverse	TGCTTGTGCAGTGTGAGAAAG
hsa-FGFR2 F	Forward	TGACCAAACGTATCCCCCTG
hsa-FGFR2 R	Reverse	TGTCTGCCGTTGAAGAGAGG
hsa-EFBN2 F	Forward	GGAAGTACTGCTGGGGTGTT
hsa-EFBN2 R	Reverse	GTACCAGTCCTTGTCCAGGTAG
hsa-VEGFα F	Forward	GTGTGTGCCCACTGAGGAGT
hsa-VEGFα R	Reverse	TGTTGTGCTGTAGGAAGCTCA
hsa-FZD2 F	Forward	CCCGACTTCACGGTCTACAT
hsa-FZD2 R	Reverse	TCCACGAGTGCAGCGTCT
hsa-LRP6 F	Forward	TTTATGCAAACAGACGGGACTT
hsa-LRP6 R	Reverse	GCCTCCAACTACAATCGTAGC
hsa-AVCR1 F	Forward	CTGTTGGTCTGGTTTACTGGGA
hsa-AVCR1 R	Reverse	ATCGAGGGATCTGAAGGCAC
hsa-PYGO1 F	Forward	AGGTGTACAACTAGGAAGCCC
hsa-PYGO1 R	Reverse	TTTGGATTCGGTGGTGGAGC
hsa-RPL13a F	Forward	GCTCATGAGGCTACGGAAAC
hsa-RPL13a R	Reverse	CCGTACATTCCAGGGCAACA
Pre-microRNA primers		
hsa-premiR-329 F	Forward	AGGTTTTCTGGGTTTCTGTTTCTT
hsa-premiR-329 R	Reverse	AGGTTAACCAGGTGTGTTTCGT
hsa-premiR-410 F	Forward	AGGTTGTCTGTGATGAGTTCG
hsa-premiR-410 R	Reverse	AGGCCATCTGTGTTATATTCGT
hsa-premiR-103a1 F	Forward	ACAGTGCTGCCTTGTTGCATA
hsa-premiR-103a1 R	Reverse	CCTGTACAATGCTGCTTGATCC
hsa-premiR-103a2 F	Forward	ACAGTGCTGCCTTGTAGCAT
hsa-premiR-103a2 R	Reverse	ACAATGCTGCTTGACCTGAA
hsa-premiR-423 F	Forward	GGCAGAGAGCGAGACTTTTC
hsa-premiR-423 R	Reverse	CCTCAGACCGAGCTTTTGGAA
hsa-premiR-433 F	Forward	AGAGGCTAGATCCTCTGTGTTG
hsa-premiR-433 R	Reverse	GAGCCCATCATGATCCTTCTCA
hsa-premiR-126 F	Forward	TACTTTTGGTACGCGCTGTGA
hsa-premiR-126 R	Reverse	ACTCACGGTACGAGTTTGAAGT

hsa-premiR-487b F	Forward	TCCCTGTCCTGTTCGTTTTG
hsa-premiR-487b R	Reverse	AAGTGGATGACCCTGTACGAT
hsa-premiR-494 F	Forward	AGGTTGTCCGTGTTGTCTTC
hsa-premiR-494 R	Reverse	AGGTTTCCCGTGTATGTTTCATC
hsa-premiR-223 F	Forward	TGACAAGCTGAGTTGGACACT
hsa-premiR-223 R	Reverse	AACTGACACTCTACCACATGGA
hsa-premiR-485 F	Forward	GAGGCTGGCCGTGATGAA
hsa-premiR-485 R	Reverse	GCCGTGTATGACTCGCTTTG
hsa-premiR-16-1 F	Forward	AGCACGTAAATATTGGCGTTAAG
hsa-premiR-16-1 R	Reverse	CAGCAGCACAGTTAATACTGGA
hsa-premiR-136 F	Forward	ACTCCATTTGTTTTGATGATGGATTCT
hsa-premiR-136 R	Reverse	CATTTGAGACGATGATGGAGCA
hsa-premiR-30d F	Forward	TGTAAACATCCCCGACTGGA
hsa-premiR-30d R	Reverse	AGCTGTGTCTTACAGCTTCC
hsa-premiR-10b F	Forward	ACCCTGTAGAACCGAATTTGTG
hsa-premiR-10b R	Reverse	TCCCCTAGAATCGAATCTGTGACTA
hsa-premiR-539 F	Forward	ATCCTTGGTGTGTTCGCTTT
hsa-premiR-539 R	Reverse	CTCAAAAAGAAATTGTCCTTGTATGATT
Pri-microRNA primers		
hsa-premiR-494 F	Forward	CGAAGGAGAGGTTGTCCGTG
hsa-premiR-494 R	Reverse	AAGGCTGCATCAGGAACAGG
hsa-premiR-329-1 F	Forward	TGGGGAAGAATCAGTGGTGT
hsa-premiR-329-1 R	Reverse	GACCAGAAGGCCTCCAAGAT
hsa-premiR-329-2 F	Forward	TGTCAAGTTTGGGGAAGGAA
hsa-premiR-329-2 R	Reverse	GACCAGAAGGCCTCCAAGAT
hsa-premiR-487b F	Forward	TGGCTTTCTTTTCCGTGCTAA
hsa-premiR-487b R	Reverse	GATCCAAACACAGGAAATCGAGC
hsa-premiR-433 F	Forward	TGAAGTGGGAGAAGAAGAAGAC
hsa-premiR-433 R	Reverse	CTTCATGGTGTGGTGCCG
hsa-premiR-191 F	Forward	GTGACCTGGGGGCAGGA
hsa-premiR-191 R	Reverse	ATCTGGGCCAGGGCG
Housekeeping genes primers		
hsa-U6 F	Forward	AGAAGATTAGCATGGCCCCT
hsa-U6 R	Reverse	ATTTGCGTGTCATCCTTGCG
hsa-RPL13a F	Forward	GCTCATGAGGCTACGGAAAC
hsa-RPL13a R	Reverse	CCGTACATTCCAGGGCAACA
MicroRNA primers		
**Name**	**TaqMan microRNA Assay number**	
hsa-miR-329-3p	001101	
hsa-miR-103a-3p	000439	
hsa-miR-191-5p	002299	
hsa-miR-10b-5p	002218	
hsa-miR-126-3p	002228	
hsa-miR-155-5p	002623	
hsa-miR-16-5p	000391	
hsa-miR-223-3p	002295	
hsa-miR-30d-5p	000420	
hsa-miR-423-5p	002340	

hsa-miR-136-5p	000592
hsa-miR-370-3p	002275
hsa-miR-410-3p	001274
hsa-miR-433-3p	001028
hsa-miR-485-5p	001036
hsa-miR-487b-3p	001285
hsa-miR-494-3p	002365
hsa-miR-539-3p	001286
hsa-mir-381-3p	000571
U6 snRNA	001973
SNORD44	001094

Primer sequences used in rt/qPCR experiments

Supplementary Table S2. m6A enrichment in pre-miRs.

#	Selected microRNAs	m6A immunoprecipitation using HUAFs (m6A IP/IgG IP)
		Pre-miR fold enrichment
1	**hsa-pre-miR-10b**	*n.d.*
2	**hsa-pre-miR-103a**	164.79
3	**hsa-pre-miR-485**	*n.d.*
4	**hsa-pre-miR-423**	107.77
5	**hsa-pre-miR-30d**	*n.d.*
6	**hsa-pre-miR-329**	145.86
7	**hsa-pre-miR-126**	307.43
8	**hsa-pre-miR-155**	*n.d.*
9	**hsa-pre-miR-16**	*n.d.*
10	**hsa-pre-miR-494**	*n.d.*
11	**hsa-pre-miR-487b**	*n.d.*
12	**hsa-pre-miR-381**	*n.d.*
13	**hsa-pre-miR-191**	*n.d.*
14	**hsa-pre-miR-370**	*n.d.*
15	**hsa-pre-miR-410**	28.42
16	**hsa-pre-miR-433**	160.49
17	**hsa-pre-miR-539**	*n.d.*
18	**hsa-pre-miR-223**	*n.d.*
19	**hsa-pre-miR-136**	*n.d.*

Precursor microRNA (pre-miR) enrichment in the m6A immunoprecipitated fraction (m6A IP) relative to the negative control fraction (IgG IP). The fold enrichment was calculated using the averages of each fraction after measuring them in triplicate. HUAF, human umbilical arterial fibroblasts; n.d., not detected

Supplementary Table S3. M6A motifs in microRNA sequences.

Name	microRNA sequence	DRACH motif [1,2]	ADRA motif [3]	WKKAYK motif [4]	FIRE motif [5]
hsa-miR-423-5p	UGAGGGGCAGAGAGCG**AGACU**UU	✓			
hsa-miR-30d-5p	UGU**AAACA**UCCCCGACUGGAAG	✓			
hsa-miR-329-3p	AACACACCUGGU**UAACC**UCUUU	✓			
hsa-miR-126-3p	TCGTACCGTGAGTA**ATAA**TGCG		✓		
hsa-miR-155-5p	UUAAUGCUAAUCGUGAUAGGGGUU				
hsa-miR-16-5p	UAGCAGCACGUAAAUAUUGGCG				
hsa-miR-494-3p	UG**AAACA**UACACGGG**AAACC**UC	✓			
hsa-miR-370-3p	GCCUGCUGGGGUG**GAACC**UGGU	✓			
hsa-miR-410-3p	AAU**AUAACA**CAGAUGGCCUGU	✓	✓		
hsa-miR-539-3p	AUCAUACA**AGGACA**AUUUCUUU	✓	✓		
hsa-miR-223-3p	UGUCAGUUUGUCAAAUACCCCA				
hsa-miR-381-3p	UAUACAAGGGCAAGCUCUCUGU				
hsa-miR-10b-5p	TACCCTGT**AGAACC**GAATTTGTG	✓	✓		
hsa-miR-103a-3p	AGCAGCATTGTACAGGGCT**ATGA**		✓		
hsa-miR-191-5p	CAACGGAATCCC**AAAA**GCAGCTG		✓		
hsa-miR-136-5p	ACTCCATTTGTT**TTGATGATG**GA		✓	✓	
hsa-miR-433-3p	ATC**ATGATG**GGCTCCTCGGTGT		✓	✓	
hsa-miR-485-5p	AGAGGCTGGCCGTG**ATGA**ATTC		✓		
hsa-miR-487b-3p	AATCGTACAGGGTCATCCACTT				

Prediction of m6A location on microRNA and premature microRNA sequences based on the 4 known m6A motifs. We used the sequences of our vasoactive microRNAs from miRBase (http://www.miRbase.com) and analysed these sequences for [AGU][AG]AC[ACU] (DRACH motif), A[A/G/U][A/G]**A** (ADRA motif), [A/U][G/U][G/U]**A**[C/U][G/U] (WKKAYK motif), and UG**A**C (FIRE motif). The different motifs are underlined when present in de microRNA sequence and the predicted m6A location is marked by **A**.

Supplementary Table S4. Methylated and unmethylated microRNA miR-494-3p mimic sequences

Name	Sequence (5′ -> 3′)
hsamiR494-3p_ac	UGAAACAUACACGGGAAACCUC
hsamiR494-3p_ac_1	UG[m6A]AACAUACACGGGAAACCUC
hsamiR494-3p_ac_2	UGAA[m6A]CAUACACGGGAAACCUC
hsamiR494-3p_pa	GGUUUCCCGUGUAUGUUUCAUU

Sequences of miR-494-3p mimics. Unmethylated miR-494-3p mimic (hsamiR494-3p_ac + hsamiR494-3p_pa), m6A 3rd position [m6A1]miR-494-3p mimic (hsamiR494-3p_ac1 + hsamiR494-3p_pa), m6A 5th position [m6A2]miR-494-3p mimic (hsamiR494-3p_ac1 + hsamiR494-3p_pa); ac, active strand. pa, passive strand. [m6A], N-6-methyladenosine

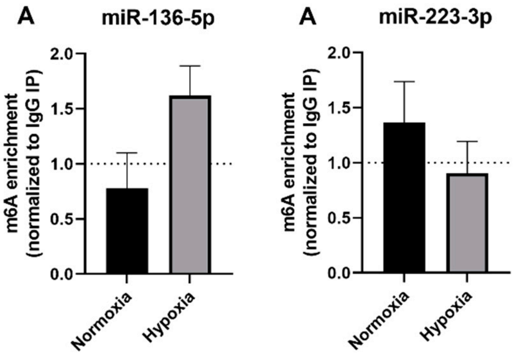

Supplemental Figure S1. No m6A enrichment in mature microRNAs miR-136-5p and miR-223-3p. Enrichment of vasoactive microRNA after m6A immunoprecipitation on small RNAs from fibroblasts cultured under hypoxic conditions for 24h or control conditions, relative to the expression in the negative control immunoprecipitation (IgG IP) in (A) miR-136-5p and (B) miR-223-3p. Results represent three independent experiments.

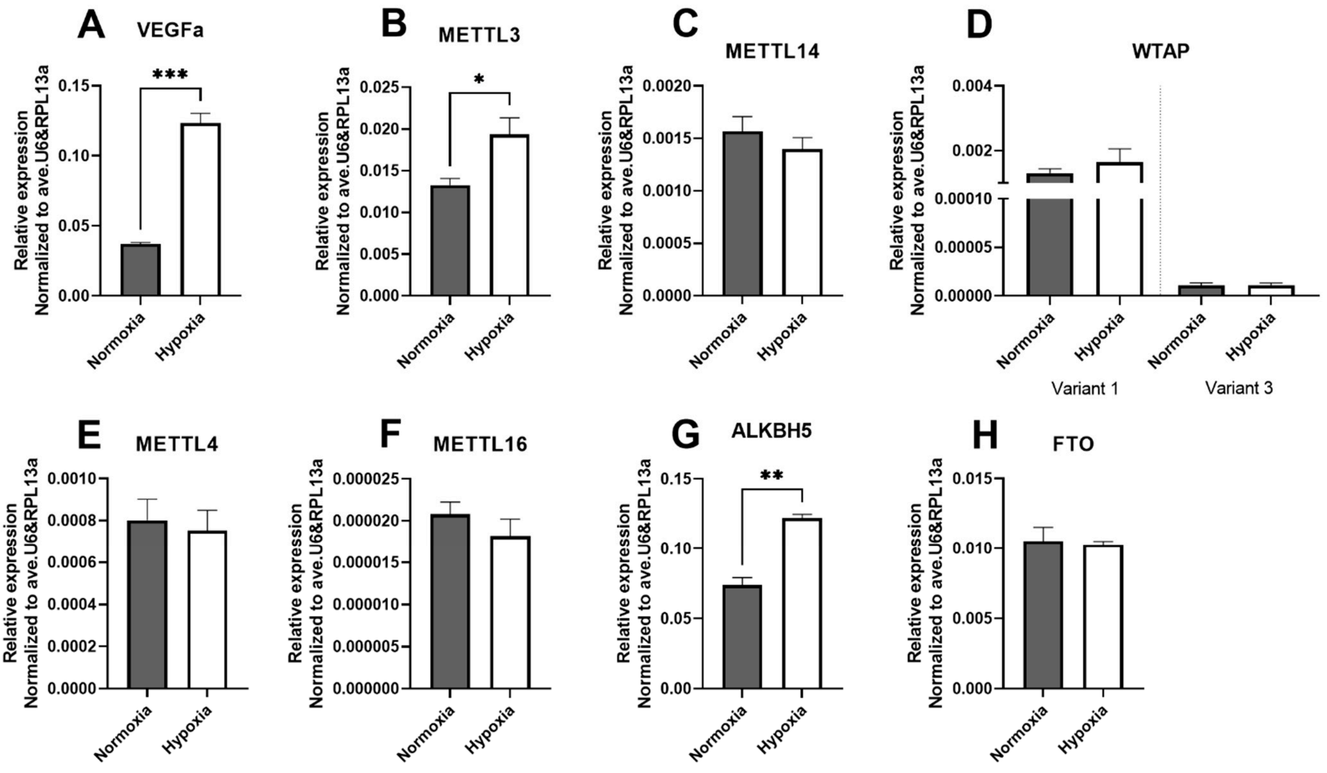

Supplemental Figure S2. Expression of m6A machinery under 48 hours of hypoxia. Relative mRNA expression levels of VEGFα (A), METTL3 (B), METTL14 (C), WTAP (D), METTL4 (E), METTL16 (F), ALKBH5 (G) and FTO (H) in BJ cells cultured under 48 hours of hypoxic conditions. All mRNA levels were normalized to an average of the housekeeping genes U6 and RPL13a. Statistically significant differences are indicated by * P≤0.05, ** P ≤ 0.01, *** P ≤ 0.001. Results represent three independent experiments.

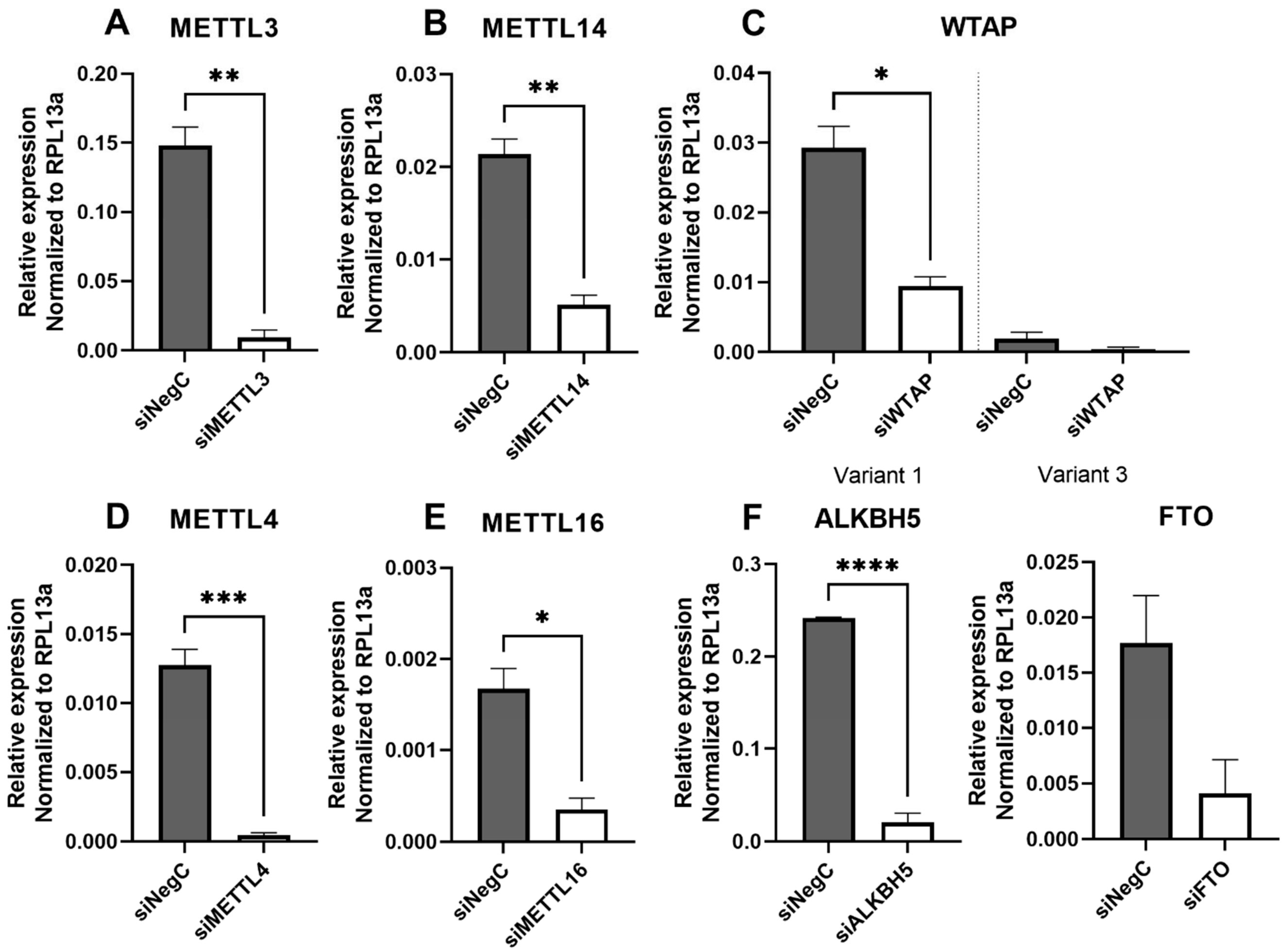

Supplemental Figure S3. Effect of siRNA on expression of m6A machinery. Relative mRNA expression levels of METTL3 (A), METTL14 (B), WTAP (C), METTL4 (D), METTL16 (E), ALKBH5 (F) and FTO (G) in BJ cells. All mRNA levels were normalized to RPL13a. Statistically significant differences are indicated by * P≤0.05, ** P ≤ 0.01, *** P ≤ 0.001, **** P ≤ 0.0001. Results represent three independent experiments.

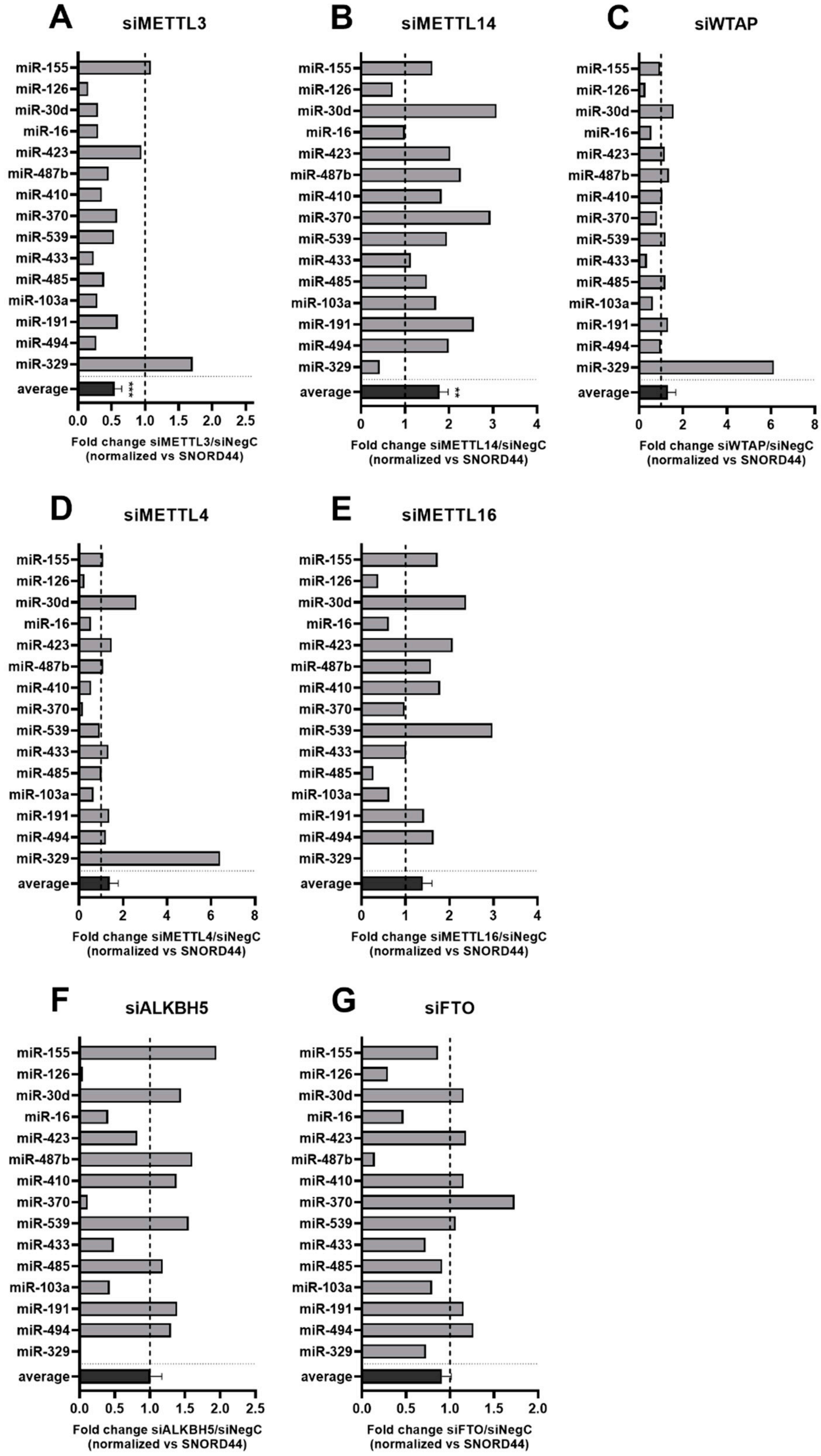

Supplemental Figure S4. Relative microRNA expression after knockdown of the m6A machinery. siRNA-induced fold change in expression of mature vasoactive microRNAs levels in 10% RNA input of m6A immunoprecipitation after knockdown of METTL3 (A), METTL14 (B), WTAP (C), METTL4 (D), METTL16 (E), ALKBH5 (F) and FTO (G) in BJ cells.

All microRNA levels were normalized to SNORD44. Statistically significant differences are indicated * $P \leq 0.05$, ** $P \leq 0.01$, *** $P \leq 0.001$.

Supplemental References

1. Dominissini, D.; Moshitch-Moshkovitz, S.; Schwartz, S.; Salmon-Divon, M.; Ungar, L.; Osenberg, S.; Cesarkas, K.; Jacob-Hirsch, J.; Amariglio, N.; Kupiec, M.; et al. Topology of the human and mouse m6A RNA methylomes revealed by m6A-seq. *Nature* **2012**, *485*, 201-206, doi:10.1038/nature11112.
2. Meyer, K.D.; Saletore, Y.; Zumbo, P.; Elemento, O.; Mason, C.E.; Jaffrey, S.R. Comprehensive analysis of mRNA methylation reveals enrichment in 3' UTRs and near stop codons. *Cell* **2012**, *149*, 1635-1646, doi:10.1016/j.cell.2012.05.003.
3. Berulava, T.; Rahmann, S.; Rademacher, K.; Klein-Hitpass, L.; Horsthemke, B. N6-adenosine methylation in MiRNAs. *PLoS One* **2015**, *10*, e0118438, doi:10.1371/journal.pone.0118438.
4. Ping, X.L.; Sun, B.F.; Wang, L.; Xiao, W.; Yang, X.; Wang, W.J.; Adhikari, S.; Shi, Y.; Lv, Y.; Chen, Y.S.; et al. Mammalian WTAP is a regulatory subunit of the RNA N6-methyladenosine methyltransferase. *Cell Res* **2014**, *24*, 177-189, doi:10.1038/cr.2014.3.
5. Alarcon, C.R.; Lee, H.; Goodarzi, H.; Halberg, N.; Tavazoie, S.F. N6-methyladenosine marks primary microRNAs for processing. *Nature* **2015**, *519*, 482-485, doi:10.1038/nature14281.
